# Supplementary material for: CDK1 phosphorylates WRN at collapsed replication forks
Source: Nat Commun. 2016 Sep 16;7:12880. doi: 10.1038/ncomms12880 (PMC5028418; doi:10.1038/ncomms12880)
Supplement: Supplementary Information — Supplementary Figures 1-17, Supplementary Table 1, Supplementary Methods and Supplementary References [file ncomms12880-s1.pdf]

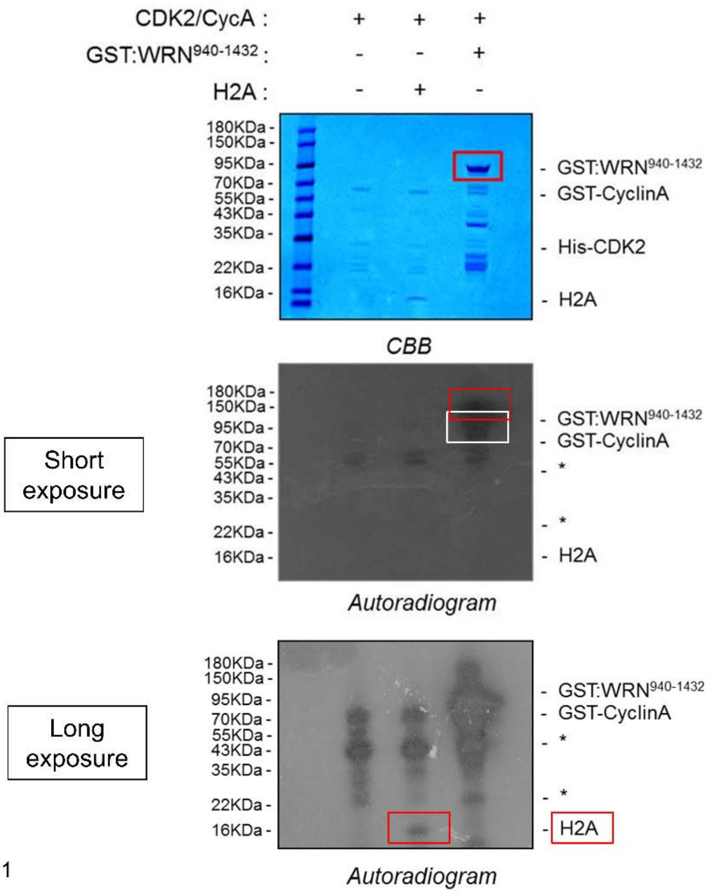

Supplementary Figure 1

Supplementary Figure 1. *In vitro* radioactive kinase assay. A GST-fused C-terminal fragment of WRN (GST-WRN$^{940\text{-}1432}$) was incubated with recombinant CDK2/CyclinA complex and radioactive ^{32}P-ATP. The recombinant histone H2A was used as positive control (Red box). The red box indicates the position of the GST-CWRN fragment in the gel. The 70KDa band phosphorylated in the autoradiogram corresponds to GST-CyclinA phosphorylated by CDK2 (white box). Asterisks denote contaminating bacterial proteins that are phosphorylated by CDK2.

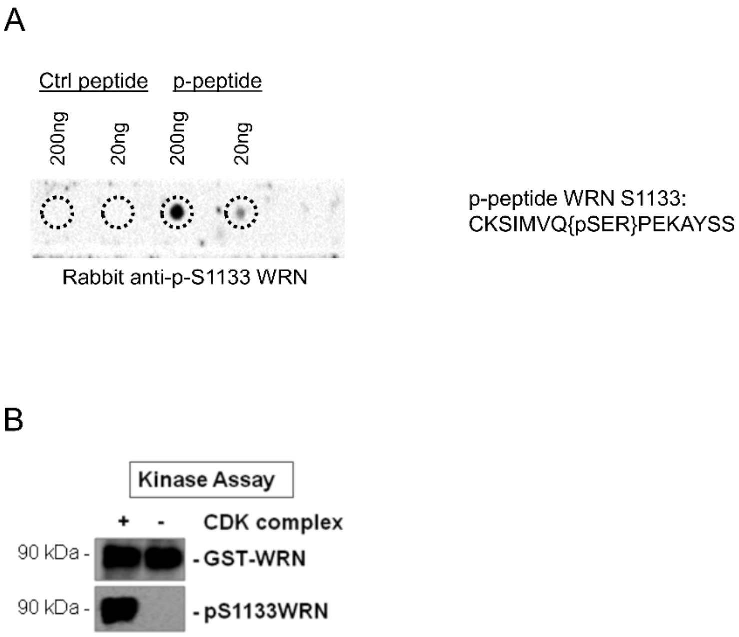

Supplementary Figure 2. A) The indicated amount of peptide or phosphopeptide containing the sequence of WRN surrounding S1133 that was used as antigen to generate the polyclonal rabbit anti-pS1133WRN antibody was spotted onto a nitrocellulose strip and subjected to WB using the pS1133WRN antibody. B) Kinase assay. The GST-fused C-terminal fragment of WRN (GST-WRN$^{940\text{-}1432}$) was incubated or not with recombinant CDK2/CyclinA complex and ATP before to be washed and resolved through SDS-PAGE. Phosphorylation at S1133 was analysed after WB with the anti-pS1133WRN antibody.

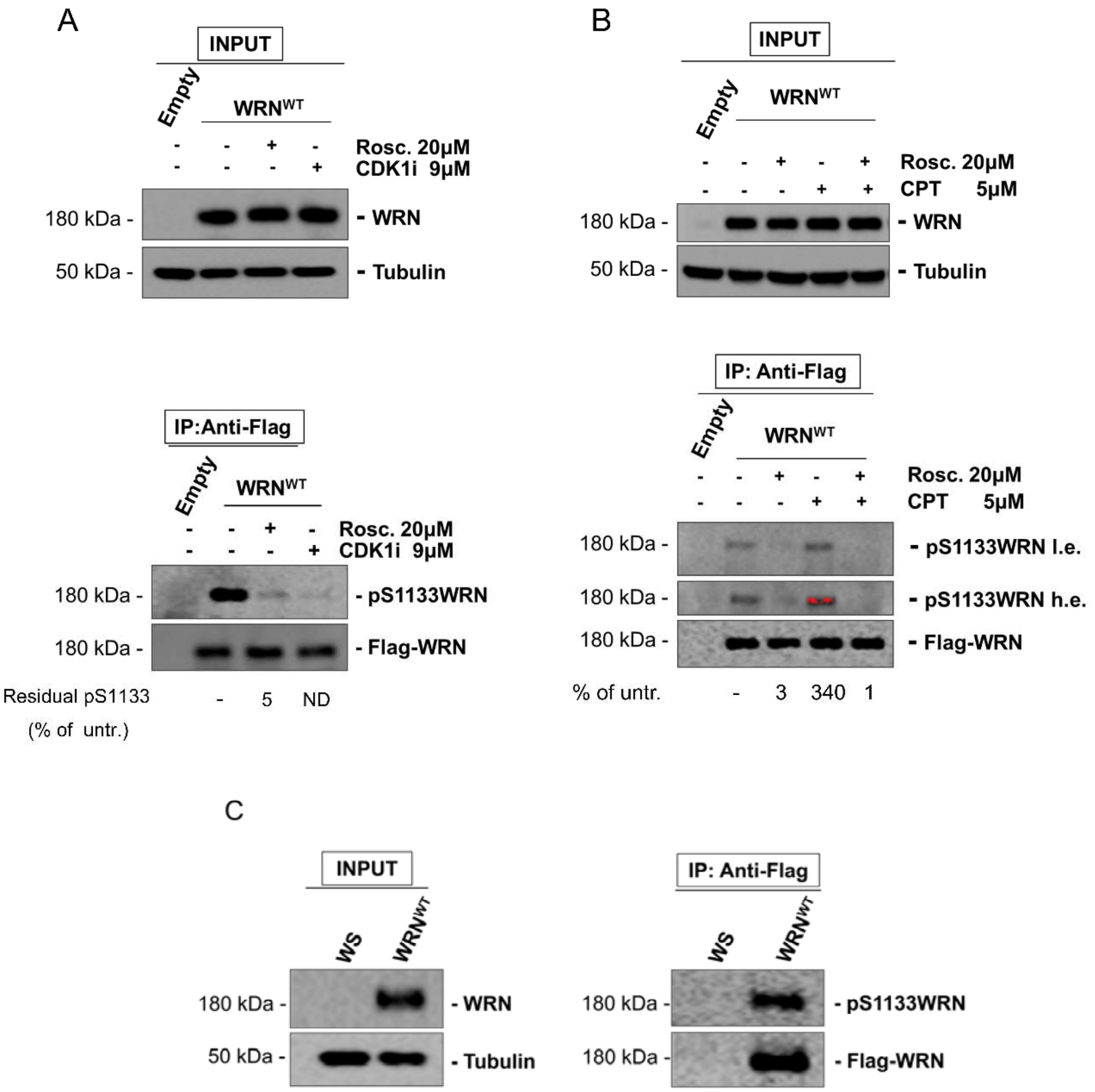

Supplementary Figure 3. CDK1 phosphorylates S1133 of WRN *in vivo*. A) The Flag-tagged WT-WRN was transiently expressed in HEK293T, and cells were treated or not with a RO-3306 (CDKi) or Roscovitine in the last 4 hrs before lysis and immunoprecipitation of WRN with anti-Flag-conjugated beads. The 9/10 of IP were subjected to SDS-PAGE and detected by WB with the anti-pS1133WRN antibody, while 1/10 was detected by anti-WRN, as indicated. One-fiftieth of the lysate (Input) was blotted to verify transfection using anti-WRN antibody. An anti-Tubulin antibody was used as loading control. Anti-Flag antibody was used to verify immunoprecipitation. B) The Flag-tagged WT-WRN or S1133A-WRN was transiently expressed in HEK293T, and cells were treated or not with CPT and Roscovitine in the last 4 hrs, as indicated. After lysis and immunoprecipitation of WRN with anti-Flag-conjugated beads, the 9/10 of IP were subjected to SDS-PAGE and detected by WB with the anti-pS1133WRN antibody, while 1/10 was detected by

anti-WRN, as indicated. One-fiftieth of the lysate (Input) was blotted to verify transfection using anti-WRN antibody. An anti-Tubulin antibody was used as loading control. Anti-Flag antibody was used to verify immunoprecipitation. C) The Flag-tagged WT-WRN was immuneprecipitated from WS cells complemented or not with WRNwt, as indicated. The 9/10 of IP were subjected to SDS-PAGE and detected by WB with the anti-pS1133WRN antibody, while 1/10 was detected by anti-WRN, as indicated. One-fiftieth of the lysate (Input) was blotted to verify transfection using anti-WRN antibody. An anti-Tubulin antibody was used as loading control. Anti-Flag antibody was used to verify immunoprecipitation.

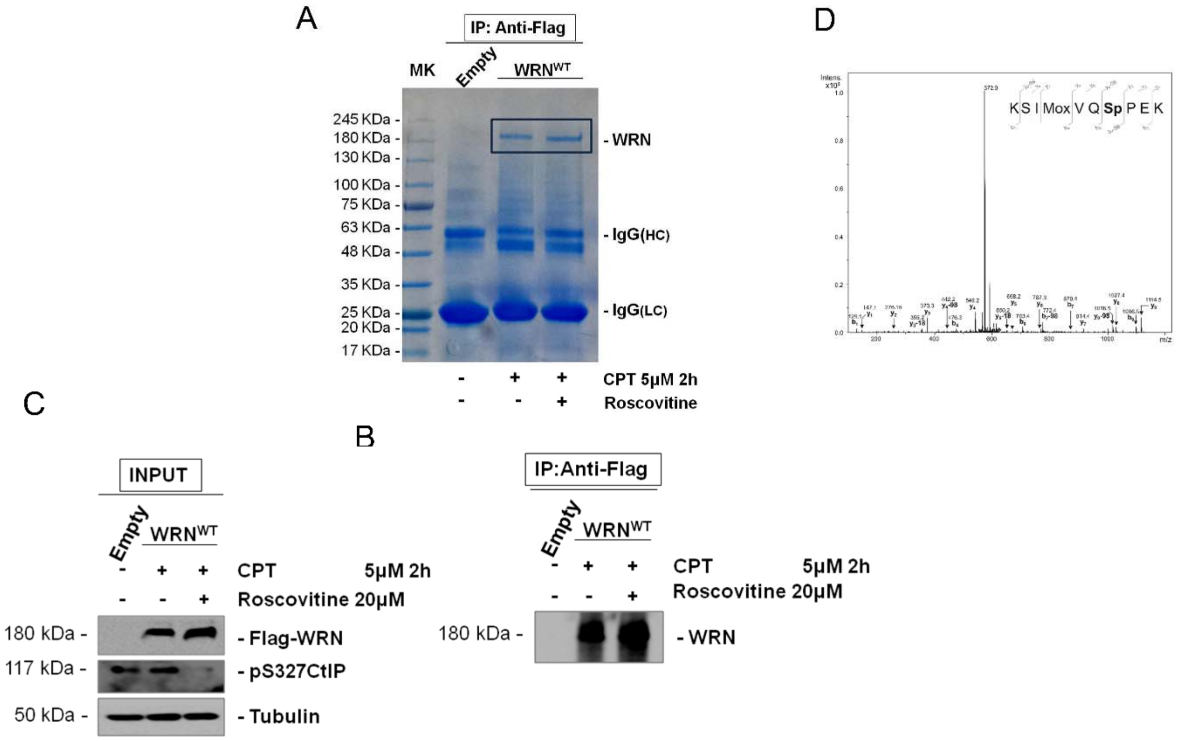

Supplementary Figure 4. Phosphorylation at S1133 of WRN can be detected after CPT treatment by Mass spectrometry. A) HEK293T cells transiently transfected with pCMV-Flag-WT-WRN were treated as indicated, lysed and Flag-WRN was immunoprecipitated (IP) with an anti-Flag antibody. IPs were separated by SDS-PAGE and the bands corresponding to WRN [black box] excised, digested, phosphopeptides enriched through TiO2 columns and analysed by Mass Spectrometry (MS). One tenth of the immunoprecipitation was analysed by WB using anti-WRN antibody to confirm identity of the 180kDa protein. C) One hundredth of the lysate used in the IP (Input), was analysed by WB using the indicated antibodies. D) The panel shows MS/MS spectrum from CPT-treated sample with identification of the WRN phosphopeptide containing S1133. Spectra from roscovitine-treated cells were not shown as S1133-containing phosphopeptides were not found after enrichment. C) WRN is phosphorylated by CDKs in vivo. HEK293T cells transiently transfected with an empty vector or a vector expressing Flag-WT-WRN were treated or not with Roscovitine for 4 hrs. WRN was immunoprecipitated (IP) with an anti-Flag antibody; 9/10 of IP were subjected to SDS-PAGE and detected by WB with the anti-pS1133WRN antibody, while 1/10 were detected by anti-WRN, as indicated. One-fiftieth of the lysate was blotted with an anti-WRN antibody to verify transfection. An anti-Tubulin antibody was used as loading control. D) Serine 1133 is targeted by CDK1. HEK293T cells transiently transfected as indicated were treated or not with RO-3306 (CDKi) for 4 hrs, immunoprecipitated (IP) with an anti-Flag antibody and subjected to WB as indicated in (C).

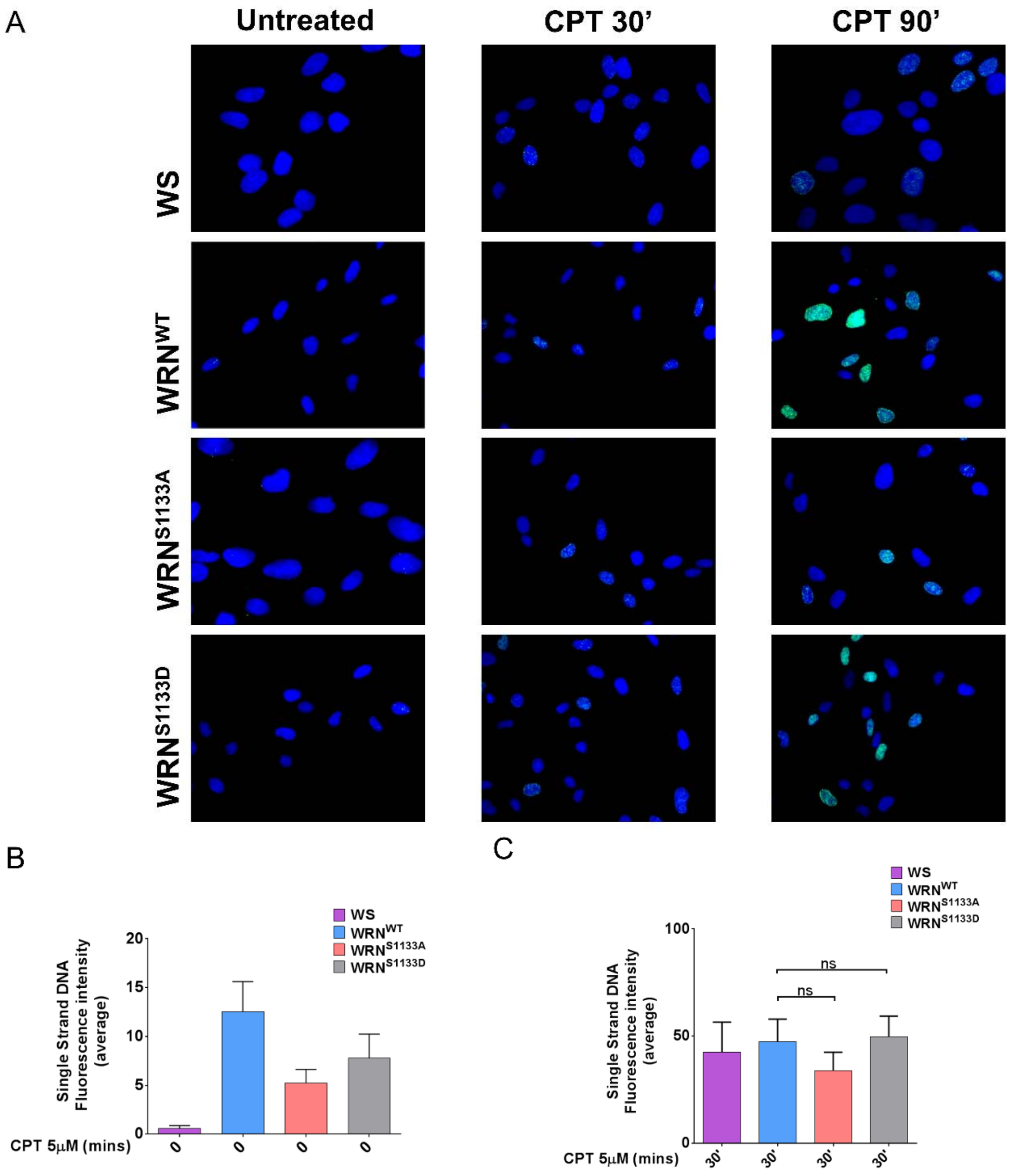

A
Untreated
CPT 30'
CPT 90'
WS
WRNWT
WRNS1133A
WRNS1133D
B
Single Strand DNA Fluorescence intensity (average)
WS
WRNWT
WRNS1133A
WRNS1133D
20
15
10
5
0
CPT 5μM (mins)
0
0
0
0
C
Single Strand DNA Fluorescence intensity (average)
100
50
0
ns
ns
CPT 5μM (mins)
30'
30'
30'
30'

D

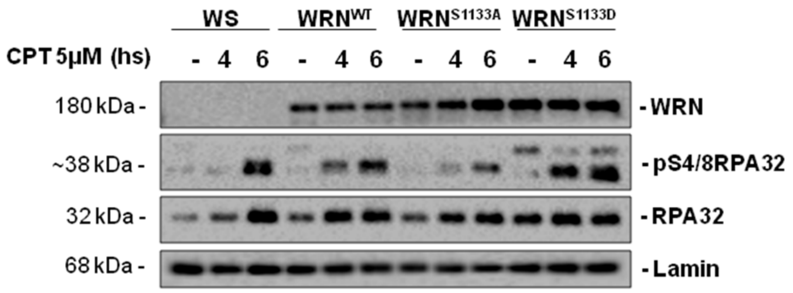

E

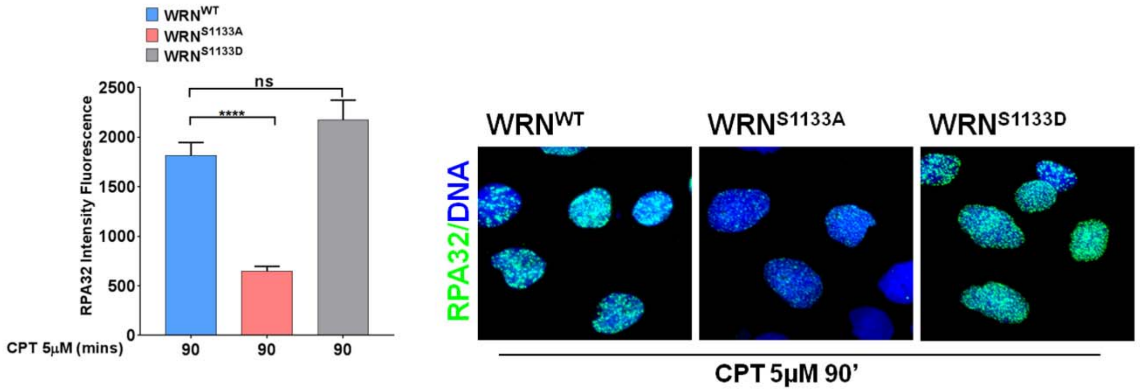

Supplementary Figure 5. A) Representative images (fields) of ssDNA detection after exposure to CPT by non-denaturing IdU immunofluorescence. WS-derived cells stably expressing the WRN wild-type (WRN^{WT}), the unphosphorylable (WRN^{S1133A}) or phosphomimetic (WRN^{S1133D}) mutant were labelled for 15 min with IdU and then treated with CPT 5µM at different time points as indicated, in the presence of IdU. DAPI was used to stain nuclei. B and C) Dot plots showing quantification of the intensity of ssDNA staining for single nuclei from WRN mutant expressing cells or WRN deficient cells (WS) treated or not with CPT for 30'. The intensity of the anti-IdU immunofluorescence was measured in at least 100 nuclei from two independent experiments. Data are presented as mean±SEM. D) Analysis of S4/8-RPA32 phosphorylation in chromatin fractions after CPT treatment. WS cells and WS-derived SV40-trasformed fibroblasts stably expressing the wild-type form of WRN, its unphosphorylable S1133A or its phosphomimetic S1133D mutant were treated with CPT as indicated. After biochemical fractionation, chromatin extracts were analysed by WB with the indicated antibodies. E) Cells were treated with CPT as indicated and analysed for the formation of RPA32 foci by IF. The graph shows quantification of the intensity of RPA32 foci staining for single nuclei from at least 150 cells in three independent experiments. The panel shows representative images. Data are presented as mean±SEM. Statistical analysis was performed by the ANOVA test (**** = $p<0.001$; ns = not significant).

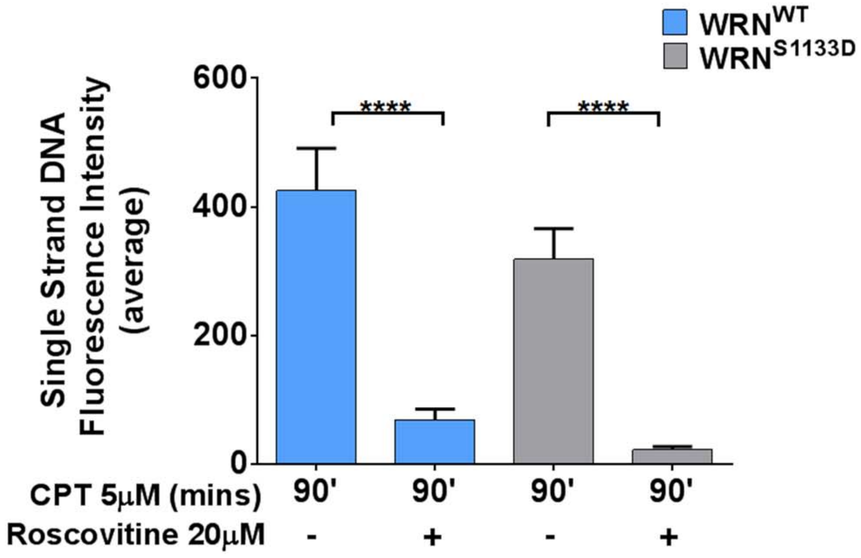

Supplementary Figure 6. Inhibition of CDKs suppresses ssDNA formation also in cells expressing the S1133D-WRN. Cells were labelled with IdU and treated with CPT. Roscovitine was added 15min before CPT treatment and kept until sampling. Graph shows the mean intensity of ssDNA staining for single nuclei from at least 100 nuclei in two independent experiments. Data are presented as mean±SEM. Statistical analysis was performed by the ANOVA test (**** = $p<0.0001$; *** = $p<0.001$; ** = $p<0.01$; * = $p<0.05$; ns = not significant).

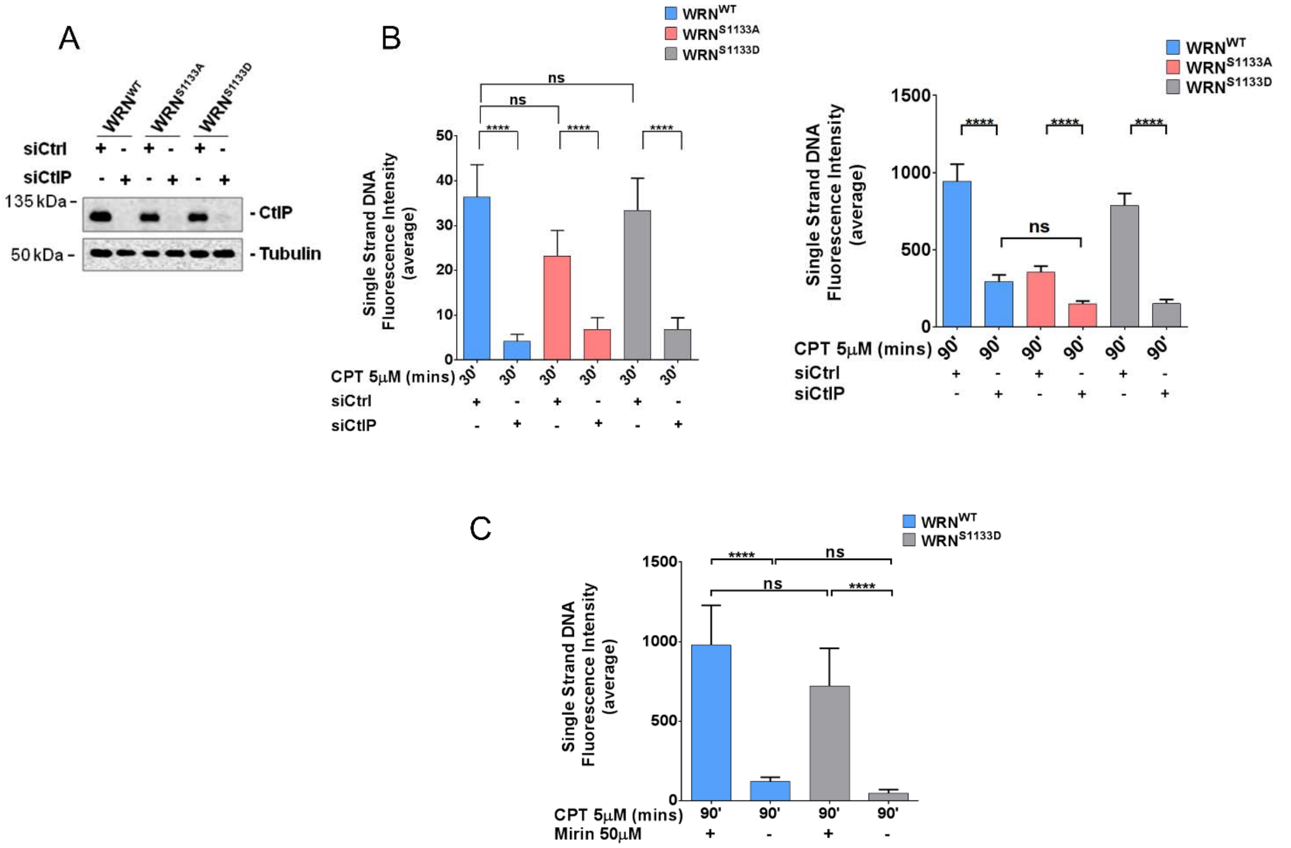

Supplementary Figure 7. CtIP depletion or MRE11 inhibition suppresses end-resection also in the presence of a phosphomimetic S1133D-WRN protein. A) Western immunoblotting showing depletion of CtIP. B) WS-derived cells were labelled and treated as in Figure 2B before ssDNA being analysed by the non-denaturing anti-IdU assay. The graph shows the mean intensity of ssDNA staining for single nuclei. The intensity of the anti-IdU immunofluorescence was measured in at least 100 nuclei from two independent experiments. C) Inhibition of MRE11 by Mirin suppresses end-resection in the presence of S1133D-WRN. Cells were labelled and treated as in Figure 2B before ssDNA being analysed by the non-denaturing anti-IdU assay. The graph shows the mean intensity of ssDNA staining for single nuclei. The intensity of the anti-IdU immunofluorescence was measured in at least 100 nuclei from two independent experiments. Statistical analysis was performed by the ANOVA test (**** = p<0.001; ns = not significant).

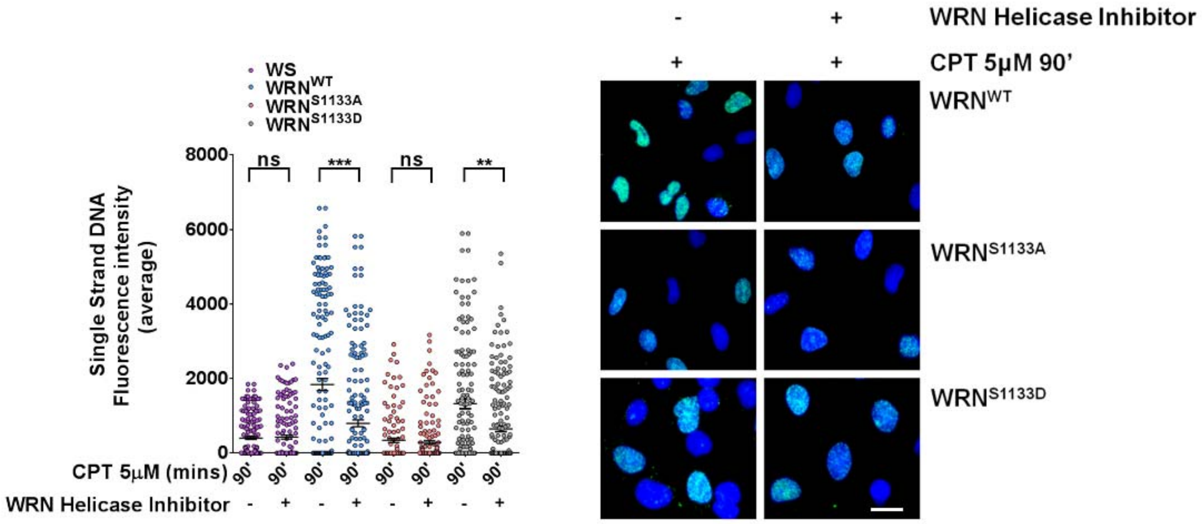

Supplementary Figure 8. The graph shows the mean intensity of IdU/ssDNA staining in cells treated or not with the WRN helicase inhibitor. The intensity of the anti-IdU immunofluorescence was measured in at least 100 nuclei from two independent experiments. The panel shows representative images of IdU/ssDNA detection after CPT treatment. Statistical analysis was performed by the ANOVA test (** = p< 0.01; *** = p<0.01; **** = p<0.001; ns = not significant).

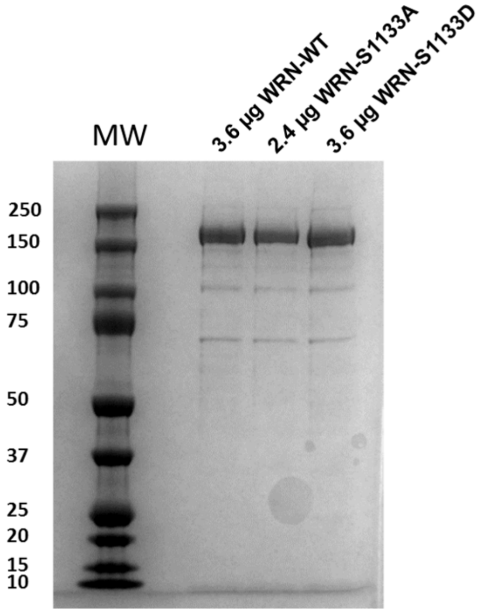

Supplementary Figure 9. Purification of recombinant Flag-WRN, Flag-WRNS1133A, and Flag-WRNS1133D. Purified recombinant WRN protein was loaded onto a 4-12% Bis-Tris NuPAGE gel (Thermo Fisher Scientific) along with protein markers (Bio-Rad).

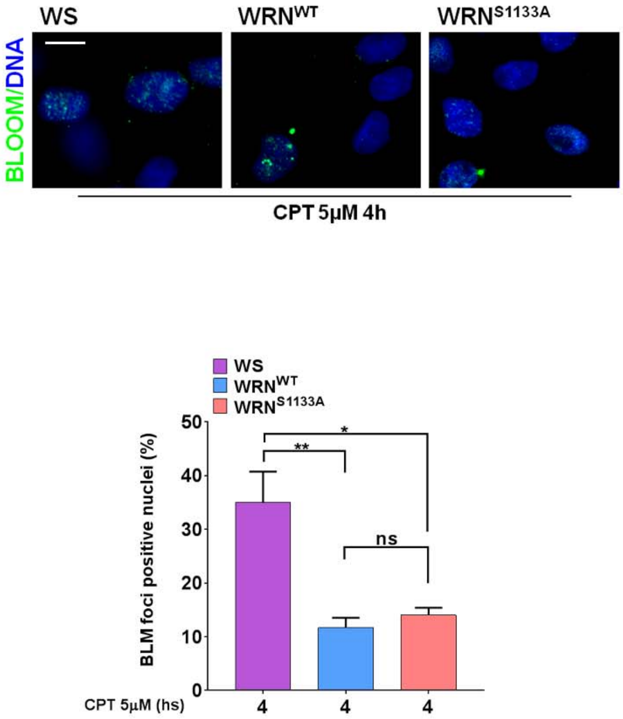

Supplementary Figure 10. Analysis of BLM-foci formation by immunofluorescence. Cells were treated as indicated. The panel shows representative images of BLM immunostaining from CPT-treated cells. DAPI was used to counterstain nuclei. The graph indicates the percentage of BLM foci-positive cells as obtained from at least 150 cells in three independent experiments. Data are presented as mean±SEM. Statistical analysis was performed by the ANOVA test (* = $p<0.05$; ** = $p< 0.01$; ns = not significant). The bar indicates 10μm.

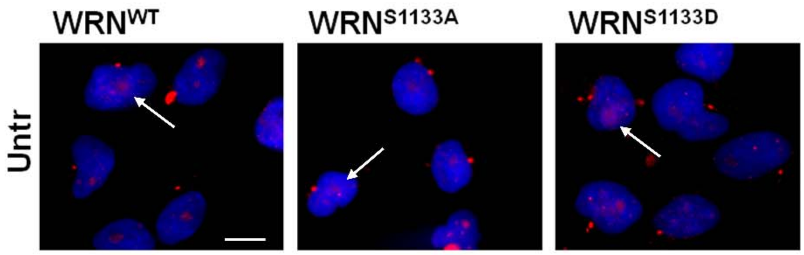

Supplementary Figure 11. Representative images of WRN immunostaining under untreated condition. DAPI was used to counterstain nuclei. The arrow indicates nucleolar localization of WRN. The bar indicates 10μm.

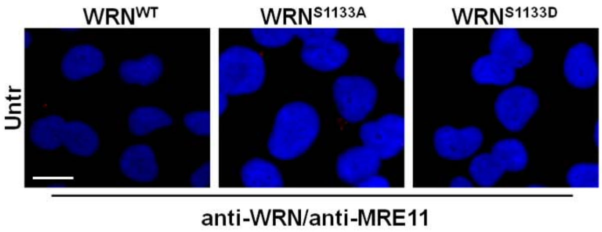

Supplementary Figure 12. The panel shows representative PLA images using anti-WRN and anti-MRE11 antibody under untreated condition. DAPI was used to counterstain nuclei. The bar indicates 10µm.

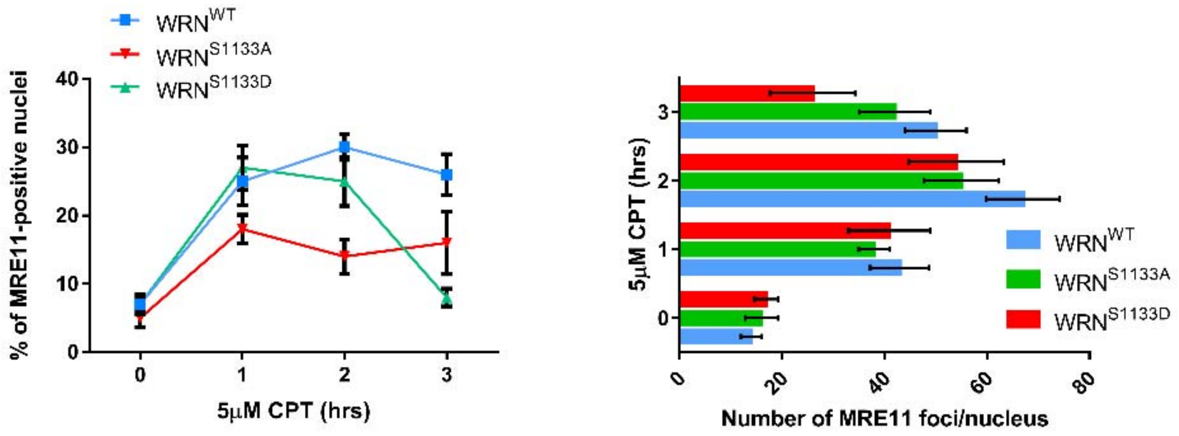

Supplementary Figure 13. WRN phosphorylation by CDK1 modulates the kinetics of formation of MRE11 nuclear foci after CPT-induced DSBs. Cells were treated or not with CPT 5μM at different time points as indicated, and the formation of MRE11 foci was evaluated by immunofluorescence. The left graph shows the percentage of MRE11-positive nuclei in the population; only nuclei with > 5 distinct foci were counted as positive. The right graph presents the count of MRE11 foci in each MRE11-positive nucleus. At least 100 nuclei were scored for each time-point. Data are presented as mean±SEM from two independent experiments.

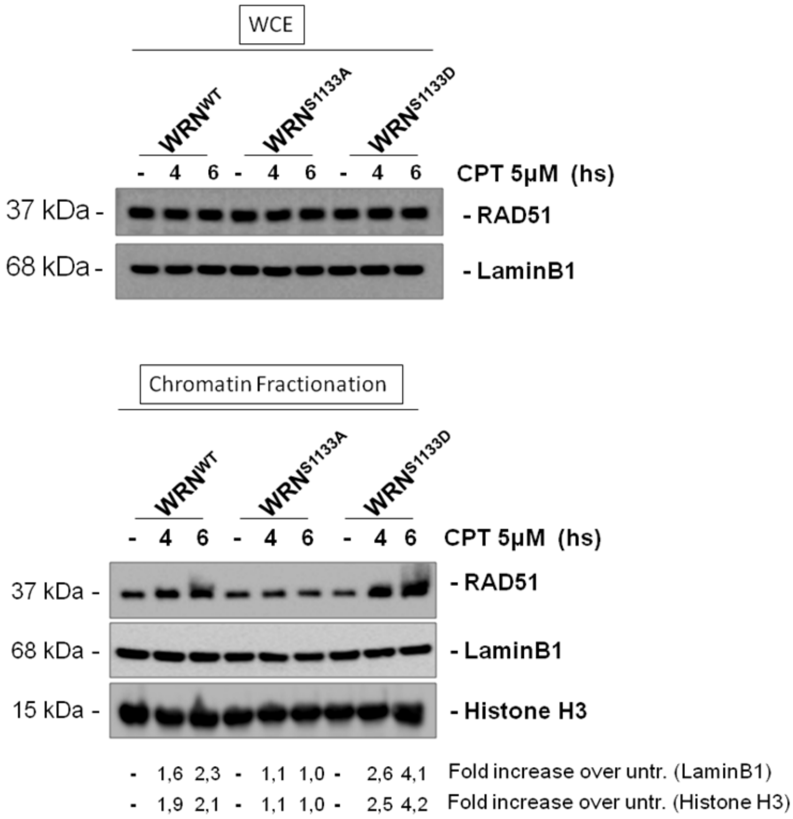

Supplementary Figure 14. Analysis of RAD51 chromatin-association after CPT treatment. Cells were treated with CPT for different time points, as indicated, and the presence of RAD51 in chromatin was assessed after cellular fractionation and Western Blotting. Anti- LaminB1 and Anti-Histone H3 were used as a loading control. F.I. = fold increase over each untreated sample after LaminB1 or Histone H3 normalization.

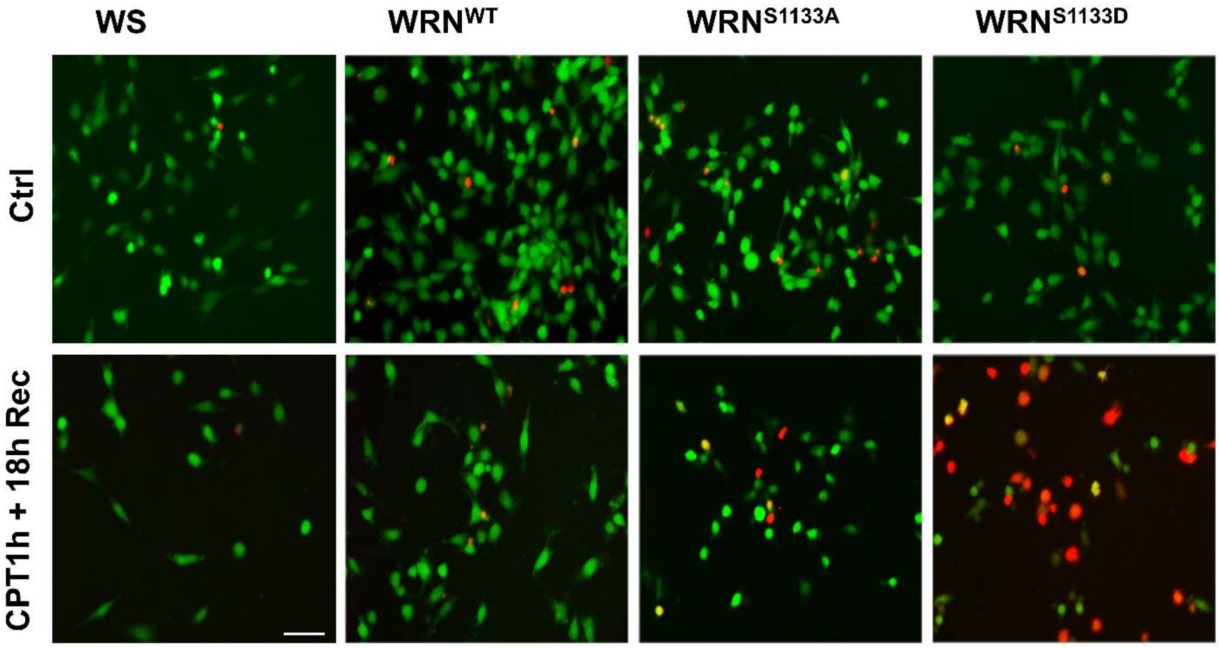

Supplementary Figure 15. Representative images of Live/Dead assay: live cells are green stained, while dead cells are red. WS cells, WS-derived cells stably expressing WRN wild-type (WRN^{WT}), the unphosphorylable (WRN^{S1133A}) or phosphomimetic (WRN^{S1133D}) WRN mutant were treated with CPT for 1 hour and recovered for 18 hours before analysis. The bar indicates 30µm.

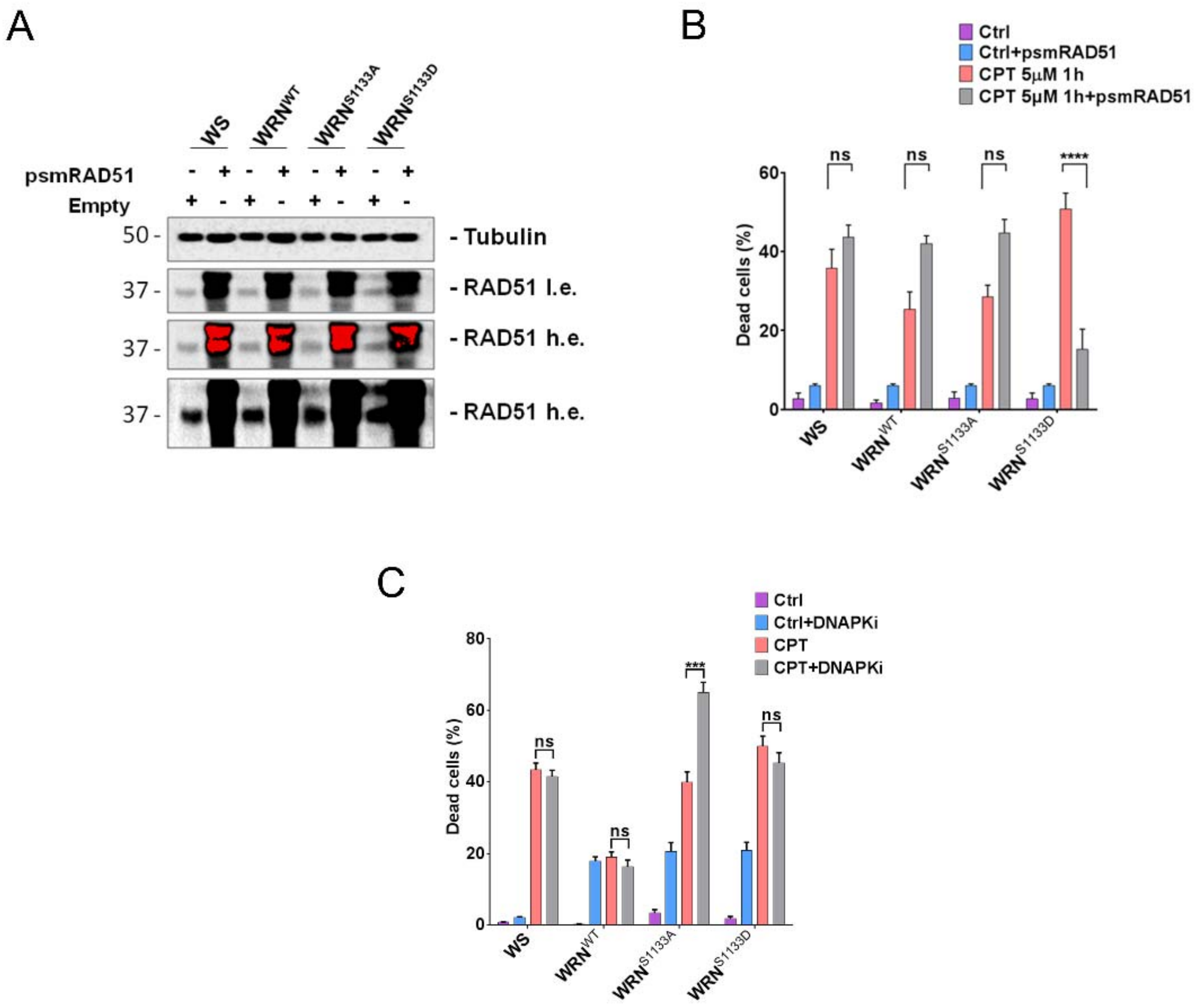

Supplementary Figure 16. Analysis of cell viability in cells transiently over-expressing a dominant-negative form of RAD51 or treated with a DNA-PKcs inhibitor. A) WB showing over-expression of the SM-RAD51 dominant-negative RAD51 chimaera. Short (l.e.) or long (h.e.) exposure of the blot is shown. Tubulin was used as loading control. B) Cells were nucleofected with the SM-RAD51 expression vector (psm-RAD51), as indicated, and 48h thereafter cell viability was evaluated by LIVE/DEAD assay, as described in materials and methods. Cells were treated with CPT for 1 h, as indicated. RAD51. Data are presented as percentage of cell death and are mean values from three independent experiments. (**** = p<0.0001; ns = not significant). C) Effect of DNA-PKcs inhibition on viability after CPT treatment. Cells were treated with CPT 5μM for 1h and recovered in drug-free medium for 18h, in the continuous presence or not of the NU-7441 inhibitor before assessing cell death by LIVE/DEAD assay. Data are presented as percentage of cell death and are mean values from three independent experiments. (*** = p<0.001, ANOVA test; ns = not significant).

Related to Fig.1C

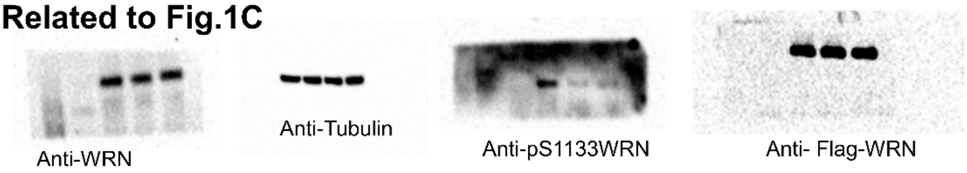

Related to Fig.1D

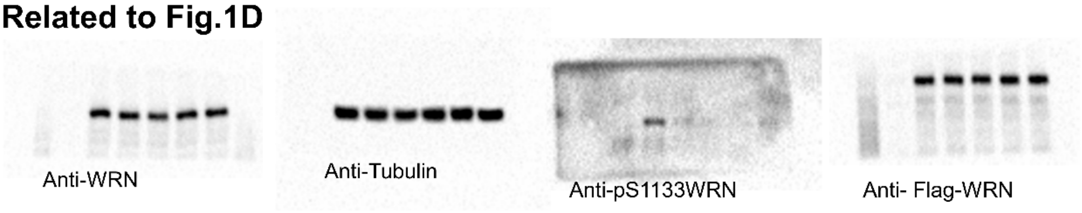

Related to Fig.2A

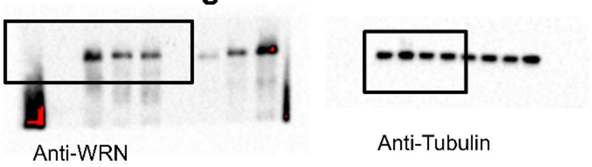

Related to Fig.4A

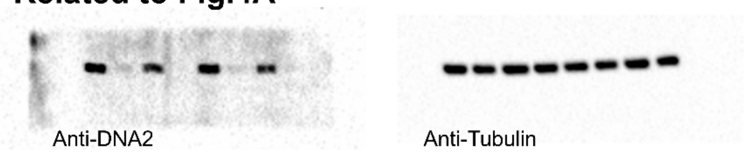

Related to Fig.4B

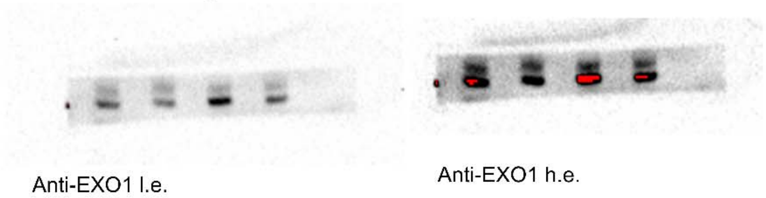

Related to Fig.6B

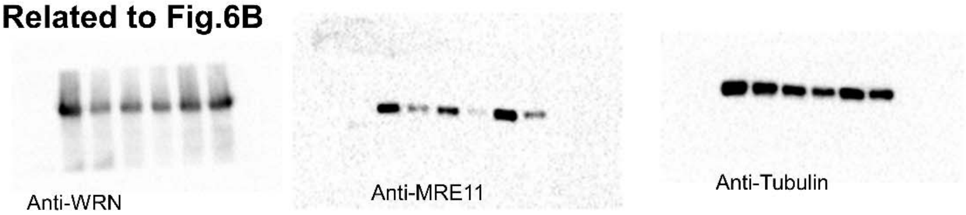

Related to Fig.7A

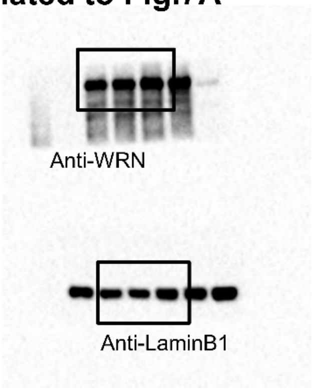

Related to Suppl. Fig.7B

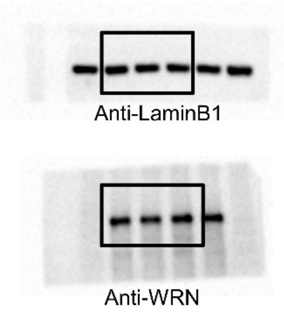

Related to Suppl. Fig.2B

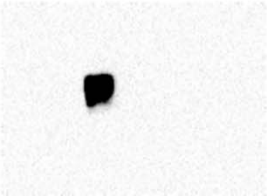
Anti-pS1133WRN

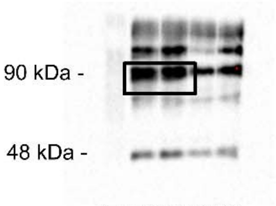

Anti-GST-WRN

Related to Suppl. Fig.3A

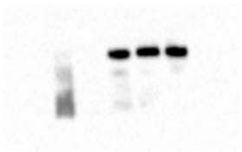
Anti-WRN

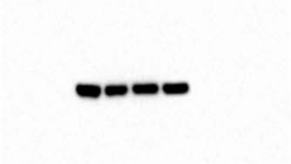
Anti-Tubulin

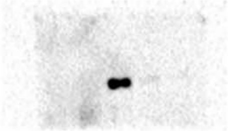
Anti-pS1133WRN

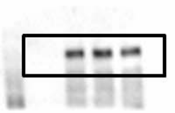
Anti- Flag-WRN

Related to Suppl. Fig.3B

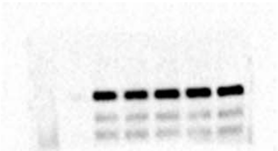
Anti-WRN

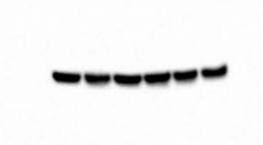
Anti-Tubulin

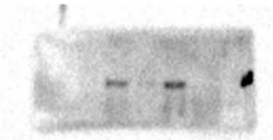
Anti-pS1133WRN l.e.

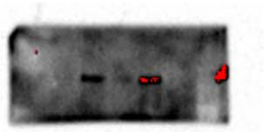
Anti-pS1133WRN h.e.

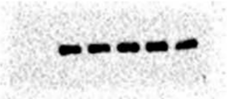
Anti- Flag-WRN

Related to Suppl. Fig.4C

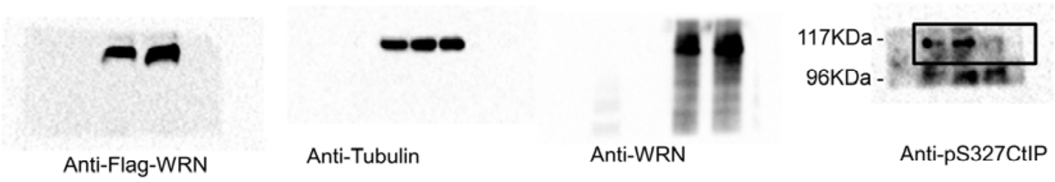

Related to Suppl. Fig.5D

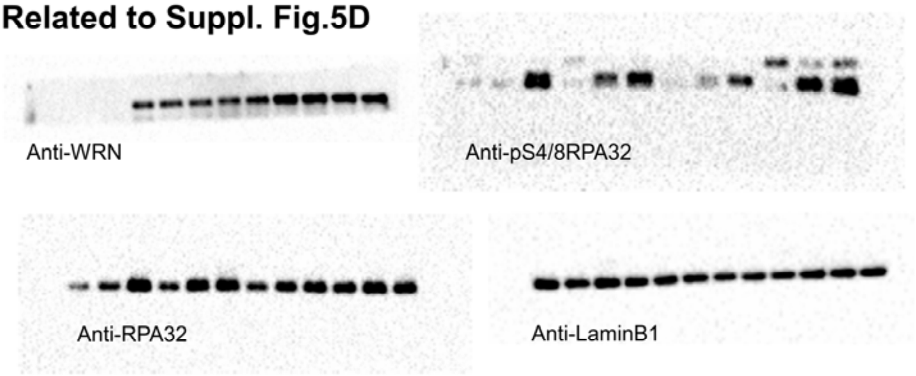

Related to Suppl. Fig.7A

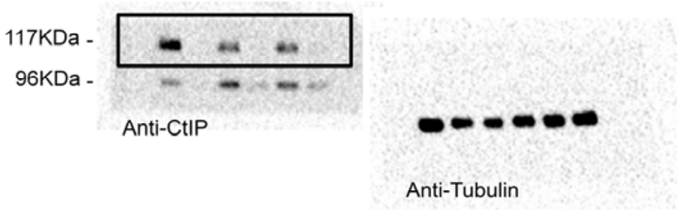

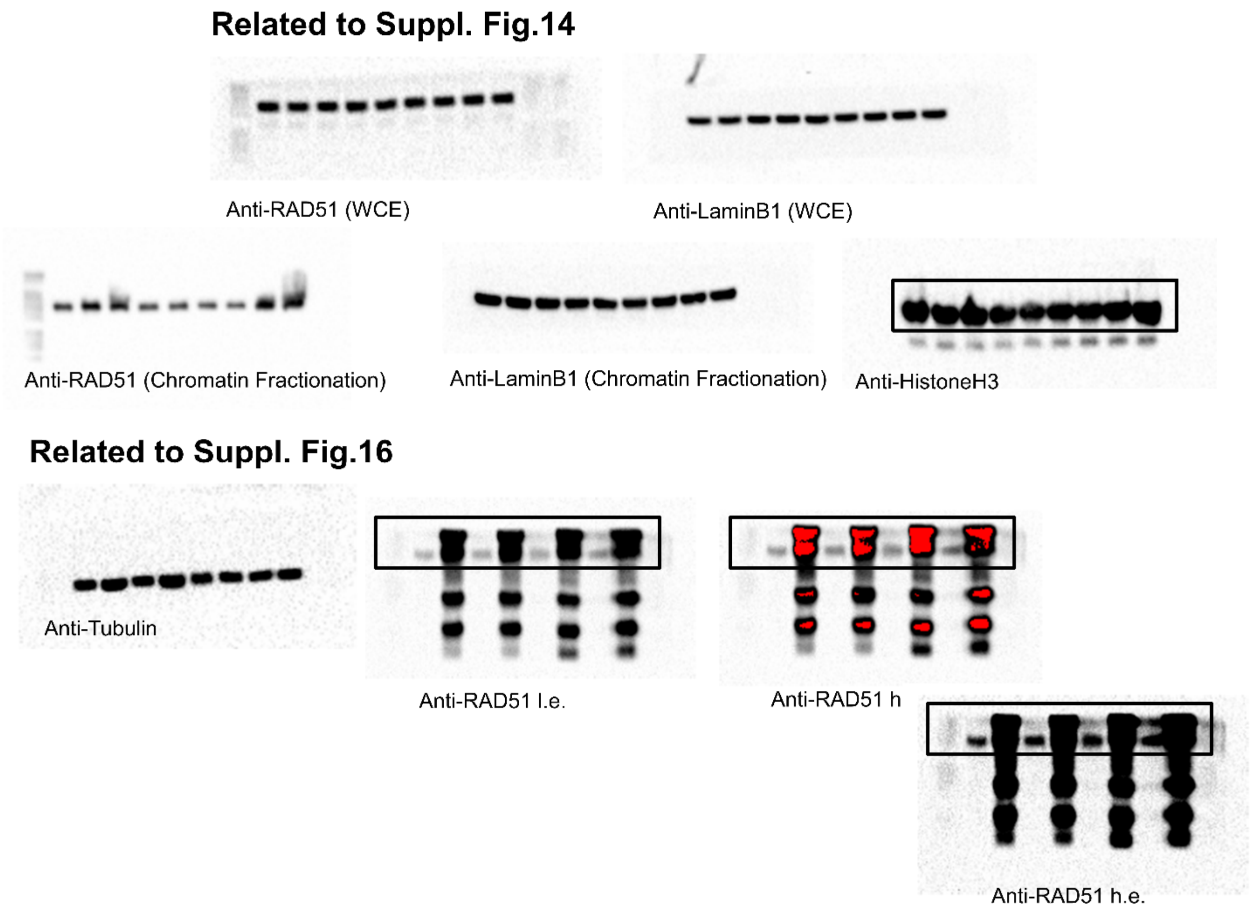

Supplementary Figure 17. Uncropped images of Western blots are presented. Reference to the original figure is presented for each blot. (l.e. = low exposure; h.e. = high exposure).

Supplementary Table 1. List of antibodies used in this study

Antibody	# Cat. Num.	Vendor	Diluition	Used for:
Rabbit Anti-WRN	# AB200	Abcam	1:2000<br>1:300	WB,<br>IF, PLA
Rabbit Anti-DNA2	# AP10182c-ev20	Abgent	1:1000	WB
Mouse Anti-Tubulin	# T0198	Sigma-Aldrich	1:3000	WB
Rabbit Anti-LaminB1	# AB16048	Abcam	1:50000	WB
Mouse Anti-DDK-Flag tag	# TA50011-100	Origene	1:2000	WB
Rabbit Anti-phosphoS4/8RPA32	# A300-245A	Bethyl	1:3000<br>1:500	WB<br>IF
Mouse Anti-RPA32	# NA19L	Calbiochem	1:2000	WB
Mouse Anti-CtIP	# SC-271339	Santa Cruz	1:500	WB
Mouse Anti-MRE11	# AB214	Abcam	1:1000<br>1:300	WB<br>IF, PLA
Rabbit Anti-RAD51	# SC-8349	Santa Cruz	1:1000	WB
Rabbit Anti-pS1133WRN	# Custom	GenScript	1:1000	WB
Rabbit Anti-GST	# T0198	Calbiochem	1:1000	WB
Rabbit Anti-pS327CtiP	# PA5-37337	Thermo Fisher Scientific	1:500	WB
Rabbit Anti-Histone H3	# NB500-171	Novus	1:1000	WB
Goat Anti-EXO1	# SC-19941	Santa Cruz	1:500	WB
Goat Anti-BLM	# SC-7790	Santa Cruz	1:50	IF
Mouse Anti-BrdU (Bromodeoxyuridine)- (Anti-IdU)	# 347580	Becton Dickinson	1:50	Fibers and ssDNA assays
Rat Anti-BrdU –(Anti-CldU)	# AB6326	Abcam	1:60	Fibers assay

SUPPLEMENTARY METHODS

In-gel digestion

Gel bands were carefully excised from the gel and subjected to in-gel trypsin digestion according to Shevchenko et al.[1] with minor modifications. The gel pieces were swollen in a digestion buffer containing 50 mM NH4HCO3 and 12.5 ng/mL trypsin (modified porcine trypsin, sequencing grade, Promega, Madison, WI) in an ice bath. After 30 min, the supernatant was removed and discarded; then 20 mL of 50 mM NH4HCO3 was added to the gel pieces, and digestion was allowed to proceed overnight at 37 °C. The supernatant containing tryptic peptides was dried by vacuum centrifugation.

Phosphopeptide enrichment

Purification of phosphopeptides was then performed according to Thingholm et al [2]. Briefly, tryptic peptides were diluted 5-fold in dihydroxybenzoic acid (DHB) buffer [350 mg/mL DHB, 80% (v/v) ACN, 2% (v/v) TFA] and applied to TiO2 beads (200 µg) pre-equilibrated in 50% ACN. The sample was then washed once in DHB buffer, before being washed two times with wash buffer [80% ACN (v/v), 2% TFA (v/v)] to remove the DHB. The sample was finally eluted with 25 µL of 2.5% ammonium hydroxide solution (pH≥10.5) and immediately neutralized with 2.5 µL of formic acid. All buffers used ultrapure water and were made fresh on the day of experimentation.

LC–ESI–CID/ETD–MS/MS

The TiO2-enriched samples were analyzed using a split-free nano-flow liquid chromatography system (EASY-nLC II, Proxeon, Odense, Denmark) coupled to a 3D-ion trap (model AmaZon ETD, Bruker Daltonik, Germany) equipped with an online ESI nano-sprayer (the spray capillary was a fused silica capillary, 0.090mm o.d., 0.020mm i.d.). A sample volume of 15 µL was loaded by the autosampler onto a homemade 2 cm fused silica precolumn (100 µm I.D.; 375 µm O.D.; Reprosil C18-AQ, 5 µm, Dr. Maisch GmbH, Ammerbuch- Entringen, Germany). Sequential elution of peptides was accomplished using a flow rate of 300 nL/min and a linear gradient from Solution A (2% acetonitrile; 0.1% formic acid) to 50% of Solution B (98% acetonitrile; 0.1% formic acid) in 40 min over the precolumn in-line with a homemade 15 cm resolving column (75 µm I.D.; 375 µm O.D.; Reprosil C18-AQ, 3 µm, Dr. Maisch GmbH, Ammerbuch-Entringen, Germany). To identify phosphorylation sites, two types of peptide fragmentation were carried out in parallel in the mass spectrometer: (i) Collision Induced Dissociation (CID); (ii) Electron Transfer Dissociation (ETD). When CID was used a MS2 was automatically performed on the three most intense MS ions, and

MS3 was triggered if one of the top three MS2 peaks corresponded with neutral loss of 98.0, 49.0, 32.7 m/z. For ETD experiments the reaction time was set to 100 ms using a reactant ICC of 500000 allowing a maximum accumulation time for the reactant ion of 10 ms. A detailed description of the acquisition parameters for the instrument was given previously [3]. Acquired MS/MS spectra were processed in DataAnalysis 4.0, and submitted to Mascot search program (in-house version 2.5, Matrix Science, London, UK). The following parameters were adopted for database searches: SwissProt database (release date 12/06/2015); taxonomy=homo sapiens; peptide mass tolerance of ±0.3 Da; fragment mass tolerance of ±0.3 for CID ions and of ±1.3 Da for ETD ions; enzyme specificity trypsin with 1 missed cleavages considered; fixed modifications: carbamidomethyl (C); variable modifications: oxidation (M), phosphorylation (STY). Phosphopeptide identifications were accepted if the Mascot score was over the 95% confidence limit based on the "identity" score of each peptide. A delta ion score was calculated of all phosphopeptides containing more than one serine, threonine or tyrosine residues by taking the difference between the two top ranking Mascot ion scores. Phosphorylation site assignments with a delta score > 5 were automatically accepted [4]. All fragmentation spectra with delta score ≤ 5 were manually inspected as to whether the phosphorylation sites were unambiguously determined or not.

Phospho-peptide antibody and Dot-blot

Phosphopeptide antibodies were raised in rabbit against the KLH- conjugated phosphopeptide (NH2)-CKSIMVQpSPEKAYSS-(COOH) for S1133 phospho-specific antibody (Genscript). Antibodies were affinity purified using phosphopeptide columns and contaminating non-phosphospecific antibody was affinity depleted by passing through a column cross-linked with non-phosphopeptide (NH2)-CKSIMVQSPEKAYSS-(COOH). The eluted phospho-specific WRN antibodies were then enriched by dialysis against TBS containing 50% glycerol and tested by the vendor. The delivered antibody showed a 100-fold higher affinity for the phosphorylated peptide as evaluated by the vendor using an ELISA plate assay. To evaluate the ability of the pS1133-WRN antibody to discriminate between unphosphorylated and phosphorylated pepetide containing S1133 in our hands, two different amounts of the peptides were spotted onto a nitrocellulose strip. After incubation for 1 h at room temperature to ensure that the blots are dry, the strip was blocked with 5% dry milk in TBS (50 mM Tris, 0.5 M NaCl, 0.05% Tween-20, pH 7.4) for 1 h at room temperature and incubate with 100ng/ml of the rabbit anti-pS1133-WRN primary antibody for 1hr at RT in 5% NFDM-TBS. After extensive washing, blot was detected with enhanced chemiluminescent reagents and images acquired through the ChemiDoc system (Bio-Rad).

Generation of the GST-WRN fragment

DNA sequence corresponding to aa 940–1432 (C-WRN) of WRN was amplified by PCR from the pCMV-FlagWRN plasmid. The PCR product were subsequently purified and sub-cloned into pGEX4T-1 vector (Stratagene) for subsequent expression in bacteria as GST-fusion proteins. The resulting vectors were subjected to sequencing to ensure that no mutations were introduced into the WRN sequence and were used for transforming BL21 cells (Stratagene). Expression of GST and GST-fusion proteins were induced upon addition of 1 mM isopropyl-D-thiogalactopyranoside (IPTG) for 2 h at 37°C. GST, and GST-C-WRN were affinity-purified using glutathione (GSH)-magnetic beads (Promega).

In vitro Kinase assay

For kinase assay, 2 μg of immunopurified GST-tagged WRN fragment was phosphorylated in vitro by CDK2-CyclinA complex (New Englad biolabs) in the presence or not of 10μCi γ32P-ATP and 5μM ATP for 30 minutes at 37°C. After the incubation, WRN fragments were separated from the beads and phosphorylation levels were assessed by SDS-PAGE followed by Coomassie staining and densitometric analysis by Phosphorimaging or by WB using anti-pS1133-WRN antibody.

Neutral comet assay

After treatment, cells were embedded in low-melting agarose and spread onto glass slides as described elsewhere [5]. DNA was stained with GelRed (Biotium) and examined at 20× magnification with an Olympus fluorescence microscope. Slides were analysed with a computerized image analysis system (Comet IV, Perceptive UK). To assess the amount of DNA DSB breaks, computer generated tail moment values (tail length × fraction of total DNA in the tail) were used. A minimum of 200 cells was analysed for each experimental point. Apoptotic cells (smaller comet head and extremely larger comet tail) were excluded from the analysis to avoid artificial enhancement of the tail moment.

RNA interference

RNAi oligos against EXO1 (an equimolar mixture of Cat. N. SI02665138—EXO1#1 and Cat. N. SI02665145—EXO1#2), and MRE11 (Cat. N. SI02665180) were from Qiagen; esiRNA (EHU070611) against CtIP were from Sigma-Aldrich. Oligos were used at the final concentration of 40, 10, 20 and 20 nM, respectively. The siRNA against DNA2 was a kind gift from Dr. Vindigni [6]. Transfection was performed using INTERFERin (Polyplus) according to the manufacturer's

instructions. As a control, a siRNA duplex directed against GFP was used. Protein down-regulation was confirmed by Western blotting 48 h after transfection.

Chromatin isolation

To isolate chromatin, cells were resuspended in buffer A (1M Hepes pH 7.9, 1M HCl, 100mM MgCl2, glycerol, sucrose, sodium fluoride, 1 mM DTT, protease inhibitors, phosphatase inhibitors). Triton X-100 (0,1%) was added, and the cells were incubated for 5 min on ice. Nuclei were collected by low-speed centrifugation (4 min, 4000 rpm, 4°C). Nuclei were washed once in buffer A, and then lysed 10 min in buffer B (3 mM EDTA, 0,2 mM EGTA, 1 mM DTT, protease inhibitors, phosphatase inhibitors). Insoluble chromatin was collected by centrifugation (4 min, 4500 rpm, 4°C), washed once in buffer B, and centrifuged again under the same conditions. The final chromatin pellet was resuspended in 2X Laemmli buffer and sonicated for 15 s in a Tekmar CV26 sonicator using a microtip at 25% amplitude. Lastly, the lysates were subjected to Western blot analysis. Control for the proper purification of the chromatin fraction, we probed blots for Lamin B1, or H3 histone which are proteins exclusively found in the chromatin fraction.

Clonogenic survival

To analyze the relative growth rate of cells expressing the wild-type or the two phosphomutant form of WRN, cells were treated with increasing doses of CPT for 24h and reseeded in 35mm dishes in triplicate at a concentration of $1x10^3$ cells/dish The number and size of methylene blue-stained colonies were recorded after 5–9 days of growth in complete medium.

Sister Chromatid Exchanges analysis

SCEs were detected as described [7]. At 24 h after seeding, cells were treated with CPT for 1h, washed in warm medium twice and recovered in the presence of 30 µM of 5-bromodeoxyuridine (BrdU; Sigma) for 36h to uniformly-label sister chromatids. Colcemid (Sigma) was added at a final concentration of 0.05 µg/ml in the last 4 h to accumulate mitotic cells prior to harvesting. Harvested cells were then incubated in hypotonic solution (0.75 M KCl) for 15 min at room temperature, and then fixed with 3:1 (vol/vol) methanol–glacial acetic acid. Fixed cell suspension was dropped onto a glass slide and air-dried. Slides were aged for 1 day and stained with 5 µg/ml Hoechst 33258 (Sigma) for 10 min. Sister chromatid differentiation was performed by the fluorescence-plus-Giemsa technique. At least 25 differentially stained metaphase chromosome spreads per treatment were scored in a blinded fashion using a bright-field microscope.

LIVE/DEAD staining

Cells were plated onto 35mm dishes, after 24h were treated or not with different drugs. Then, cells are harvested and washed once with PBS and incubated with a solution of 4 μM calcein AM (Santa Cruz) and 4 μM propidium iodide in sterile PBS for 30 min at RT. After removal of this solution, cells were fixed in 4% PFA.

Live (green) and dead (red) cells were counted using a fluorescence microscope (Zeiss) connected to CCD camera for image acquisition. Cell number was counted in random fields and expressed as percent of dead cells (number of red nuclear stained cells/total cell number). For each time point, at least 300 cells were counted.

Generation of Baculovirus

FLAG-WRN, FLAG-WRNS1133A, and FLAG-WRNS1133D were subcloned from the pFLAGWRN plasmid into pFastBacHTB construct (Thermo Fisher Scientific) via 5′ NotI and 3′ KpnI sites, making the pFastBacHTB-FLAG-WRN, pFastBacHTB-FLAG-WRNS1133A and pFastBacHTB- FLAG-WRNS1133D plasmids which were sequenced for verification. Miniprep plasmids were transformed into DH10Bac to generate baculovirus DNA containing the His-FLAG-WRN sequence. The baculovirus DNA were transfected into sf9 insect cells using Cellfectin II (Thermo Fisher Scientific) according to the manufacturer's recommendations. After five days, media was harvested and used to reinfect sf9 insect cells to generate high titer virus. After 5 days, the media containing high titer virus was collected and stored at 4° C.

Purification of recombinant FLAG-WRN, FLAG-WRNS1133A, and FLAG-WRNS1133D

High titer virus was used to infect Hi5 insect cells (Thermo Fisher Scientific) at an MOI of approximately 10. Cells were harvested 48 hours later and placed in -80° C until lysed. Cell pellets for His-FLAG-WRN, His-FLAG-WRNS1133A, and His-FLAG-WRNS1133D containing approximately 1.2×10^8 cells each were resuspended in 10 ml of Lysis Buffer (50 mM Tris pH 7.4, 150 mM NaCl, 0.4% NP40, 10% glycerol, 5 mM BME, and Complete Ultra Protease Inhibitors (Roche)), vortexed and rotated at 4° C for 45 minutes. The lysates were centrifuged at 20,000 RPM for 10 minutes and the supernatant was passed through a 0.45 μm PVDF filter. Each clarified lysate was passed twice through a Ni2+-charged 1 ml HiTrap Chelating HP column (GE Healthcare Life Sciences) which had been equilibrated in Buffer TN (50 mM Tris pH 7.4, 150 mM NaCl, 10% glycerol, 5 mM BME, protease inhibitors) with 10 mM imidazole. 5 ml washes with TN buffer containing 10 mM, 20 mM, and 40 mM imidazole each were performed followed by elution with TN buffer containing 400 mM imidazole. The eluted protein was pooled and incubated with TEV

protease for 16 hours at 4° C to cleave the 6xHis tag off the protein. The protein was dialyzed into NETN-500 Buffer (50 mM Tris pH 7.4, 500 mM NaCl, 0.5% NP40, 1 mM EDTA) using an Amicon Ultra 100 kD cutoff centrifugal filter (EMD Millipore). The retained sample was applied to 250 µl of packed M2 anti-FLAG beads (Sigma) which had been equilibrated in NETN-500 buffer. The beads were washed twice with 5 ml of NETN-500 buffer and the WRN protein was eluted with 3X FLAG peptide twice in 500 µl Storage Buffer (100 mM Tris pH 8.0, 400 mM NaCl, 10% glycerol, 5 mM BME). Eluted protein was concentrated and dialyzed against storage buffer in the absence of FLAG peptide and frozen at -80° C.

Helicase assays

The 19 base pair forked duplex DNA substrate was prepared as previously described [8]. Purified recombinant WRN protein (concentrations indicated in figure legend) or storage buffer was incubated with 0.5 nM forked DNA substrate (19 base pairs and 25 nucleotide 5′ and 3′ tails) in 20 µl reactions containing 30 mM HEPES pH 7.4, 40 mM KCl, 100 µg/ml BSA, 8 mM MgCl2, 5% glycerol, and 2 mM ATP for 15 minutes at 37° C. Reactions were stopped by adding 20 µl of 9 mM EDTA, 0.6% SDS, 0.04% bromphenol blue, 0.04% xylene cyanol, and 25% glycerol containing a 10-fold excess of the labeled oligo without the radiolabel. The heat-denatured sample was boiled for 5 minutes at 95° C. Samples were loaded onto a 12% non-denaturing gel and run at 200 V for 1.5 hours. Gels were exposed to a phosphorimager screen and imaged with a Typhoon imager.

Exonuclease assays

Purified recombinant WRN protein (concentrations indicated in figure legend) or storage buffer was incubated with 0.5 nM forked DNA substrate (19 base pairs and 25-nucleotide 5′ and 3′ tails) in 10 µl reactions containing 40 mM Tris pH 8.0, 4 mM MgCl2, 5 mM DTT, and 1 µg/ml BSA for 20 minutes at 37° C. Reactions were stopped by adding 10 µl of formamide with 0.1% xylene cyanol and 0.1% bromphenol blue and heating for 3 minutes at 95° C. Six-µl of each sample was loaded onto a pre-warmed 14% denaturing PAGE gel with 7 M Urea and run at 40 W for 45 minutes. Gels were exposed to a phosphorimager screen and imaged with a Typhoon imager.

RNAi and SDM oligonucleotides

Cat. Num. Or target sequence	Gene Name
EHU070611-20UG (Sigma-aldrich)	RBBP8 (CtIP)
SI02665138 (Qiagen)	EXO1#1
SI02665145 (Qiagen)	EXO1#2
SI02665180 (Qiagen)	MRE11
5’-CAGUAUCUCCUCUAGCUAG-3’	DNA2

SDM Primers

WRN_S1133A_FW

5’ CTAAAAAAAGTATCATGGTACAGGCACCAGAAAAAGCTTACAGTTCC 3’

WRN_S1133A_RW

5’ GGAACTGTAAGCTTTTTCTGGTGCCTGTACCATGATACTTTTTTTAG 3’

WRN_S1133D_FW

5’ ATTTCTAAAAAAAGTATCATGGTACAGGATCCAGAAAAAGCTTACAGTTCCTCACAG 3’

WRN_S1133D_RW

5’ CTGTGAGGAACTGTAAGCTTTTTCTGGATCCTGTACCATGATACTTTTTTTAGAAAT 3’

SUPPLEMENTARY REFERENCES

1. Shevchenko, A., Wilm, M., Vorm, O. & Mann, M. Mass spectrometric sequencing of proteins silver-stained polyacrylamide gels. *Anal. Chem.* **68,** 850–8 (1996).
2. Thingholm, T. E., Jørgensen, T. J. D., Jensen, O. N. & Larsen, M. R. Highly selective enrichment of phosphorylated peptides using titanium dioxide. *Nat. Protoc.* **1,** 1929–35 (2006).
3. D'Alessandro, A. *et al.* Love me tender: an Omics window on the bovine meat tenderness network. *J. Proteomics* **75,** 4360–80 (2012).
4. Wang, Y.-T. *et al.* An informatics-assisted label-free quantitation strategy that depicts phosphoproteomic profiles in lung cancer cell invasion. *J. Proteome Res.* **9,** 5582–97 (2010).
5. Ammazzalorso, F., Pirzio, L. M., Bignami, M., Franchitto, A. & Pichierri, P. ATR and ATM differently regulate WRN to prevent DSBs at stalled replication forks and promote replication fork recovery. *EMBO J.* **29,** 3156–3169 (2010).
6. Thangavel, S. *et al.* DNA2 drives processing and restart of reversed replication forks in human cells. *J. Cell Biol.* **208,** 545–62 (2015).
7. Palitti, F., Pichierri, P., Franchitto, a, Proietti De Santis, L. & Mosesso, P. Chromosome radiosensitivity in human G2 lymphocytes and cell-cycle progression. *Int. J. Radiat. Biol.* **75,** 621–627 (1999).
8. Brosh, R. M., Waheed, J. & Sommers, J. a. Biochemical characterization of the DNA substrate specificity of Werner syndrome helicase. *J. Biol. Chem.* **277,** 23236–23245 (2002).
